# Effects of sodium-glucose cotransporter-2 inhibitors and dipeptidyl peptidase-4 inhibitors on diabetic retinopathy and its progression: A real-world Korean study

**DOI:** 10.1371/journal.pone.0224549

**Published:** 2019-10-28

**Authors:** Yoo-Ri Chung, Kyoung Hwa Ha, Kihwang Lee, Dae Jung Kim

**Affiliations:** 1 Department of Ophthalmology, Ajou University School of Medicine, Suwon, Korea; 2 Department of Endocrinology and Metabolism, Ajou University School of Medicine, Suwon, Korea; 3 Cardiovascular and Metabolic Disease Etiology Research Center, Ajou University School of Medicine, Suwon, Korea; Kaohsiung Medical University Hospital, TAIWAN

## Abstract

The sodium-glucose cotransporter-2 inhibitors (SGLT2is) reduce the incidence of macrovascular complications of diabetes, while their effect on diabetic retinopathy has not been clarified. We compared the effects of SGLT2is with those of dipeptidyl peptidase-4 inhibitors (DPP4is) on the risk of diabetic retinopathy and its progression in people with type 2 diabetes. We performed a retrospective cohort study among people with type 2 diabetes who started on a SGLT2i or DPP4i from 2014 to 2016 according to the Korean National Health Insurance Service database. Subjects initiated on a SGLT2i or DPP4i were matched on a 1:1 basis according to their propensity scores, and Cox proportional hazards regression models were used to calculate the hazard ratios for the risk of diabetic retinopathy and its progression. After propensity score-matching, 41,430 patients without a history of diabetic retinopathy were identified as new users of a SGLT2i (n = 20,175) or DPP4i (n = 20,175). The hazard ratio (95% CI) for diabetic retinopathy was 0.89 (0.83–0.97) for SGLT2i initiators compared with DPP4i initiators. In patients with a history of diabetic retinopathy (n = 4,663 pairs), there was no significant difference in diabetic retinopathy progression between SGLT2i initiators and DPP4i initiators (hazard ratio 0.94, 95% CI 0.78–1.13). This real-world cohort study showed that SGLT2is might be associated with lower risk of diabetic retinopathy compared with DPP4is. Randomized controlled trials are needed to investigate the long-term effect of SGLT2is in diabetic retinopathy in people with diabetes.

## Introduction

The sodium-glucose cotransporter-2 inhibitors (SGLT2is) are a newly introduced class of anti-hyperglycemic agents that lower the blood glucose level by reducing glucose reabsorption in the renal proximal tubule [1]. They also induce weight loss and lower blood pressure; these effects have led to multiple randomized controlled trials of their influence on cardiovascular outcomes [2–4]. In particular, the use of a SGLT2i was associated with a lower risk of hospitalization for heart failure and all-cause death [5]. Recent real-world studies reported a lower risk of cardiovascular events with SGLT2i compared to other glucose-lowering drugs [6–9]. Additionally, the SGLT2i dapagliflozin had a lower risk of cardiovascular events compared to a dipeptidyl peptidase-4 inhibitor (DPP4i) [10]. However, these real-world studies did not report any data on diabetic retinopathy (DR) which is critical to visual prognosis concerning quality of life in diabetic patients [6–9].

SGLT2is reduce the incidence of not only macrovascular but also microvascular complications by affecting vascular remodeling [11, 12], and several preclinical and clinical studies have suggested renoprotective activity. This renoprotection may be due to suppression of the renin-angiotensin system, decreased inflammation and oxidative stress, decreased lipid accumulation, and restored renal hemodynamics [13–15]. DR, one of the major microvascular complications of diabetes, shares the same microvascular changes with diabetic nephropathy [16]. DR in late stages can be treated by laser photocoagulation, intravitreal anti-vascular endothelial growth factor (VEGF) agents or corticosteroids, and vitrectomy surgery, while the ability of these treatments to restore already-impaired vision is limited [17, 18]. As the pathogenesis of diabetic nephropathy and DR are similar [16], we hypothesized that SGLT2i may also protect against DR. A retrospective pilot study using the medical records of persons with type 2 diabetes showed that a SGLT2i slowed the progression of DR [19].

Accordingly, we conducted a real-world cohort study to investigate the effect of SGLT2i on the occurrence and progression of DR compared with DPP4i among people with type 2 diabetes using the Korean health insurance database.

## Materials and methods

### Data sources

The National Health Insurance Service (NHIS) of South Korea is a compulsory single-payer health insurance system that covers 98% of the population [20, 21]. The NHIS claim database includes demographic information, diagnoses, prescriptions, and procedures. The NHIS also requires all insured employees and self-employed individuals aged ≥ 40 years as well as their family dependents to undergo a national health screening examination every 2 years. This health screening includes body size, blood pressure, blood chemistry data (including fasting glucose level and lipid profile), health behaviors, and personal and family histories of disease. The study protocol was reviewed and approved by the Institutional Review Board of Ajou University Hospital, Suwon, Korea (IRB No. AJIRB-MED-EXP-17-510). Because the data in this database were de-identified, the requirement for informed consent was waived.

### Study population and drug exposure

**Fig 1:**
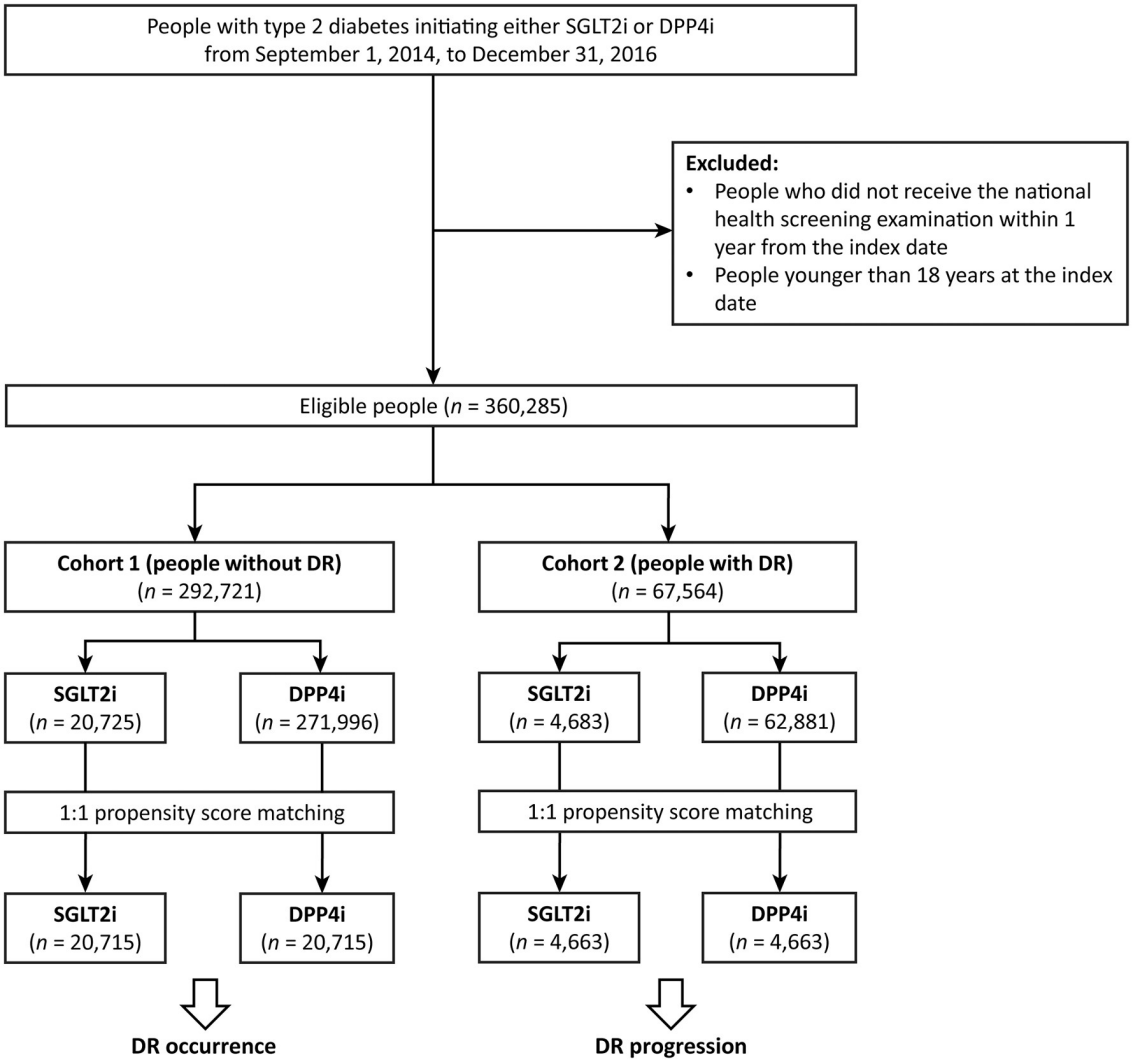
Flow chart of the study cohorts. DPP4i, dipeptidyl peptidase-4 inhibitor; DR, diabetic retinopathy; SGLT2i, sodium-glucose cotransporter-2 inhibitor.

We identified persons with type 2 diabetes (International Classification of Diseases, 10th revision [ICD-10] codes: E11 to E14) aged ≥ 18 years who initiated SGLT2i or DPP4i therapy. New users were defined as those whose date of initial exposure (index date) to a SGLT2i or DPP4i was during the study period (September 2014 to December 2016) and who had no prior exposure in the previous year. We excluded persons diagnosed with type 1 diabetes (ICD code: E10) or gestational diabetes (ICD code: O24) before the initial exposure to a SGLT2i or DPP4i, as well as those who did not undergo the national health screening examination in the year prior to the index date. The study population was divided into the following cohorts (Fig 1): subjects without a history of DR (cohort 1) and those with DR (cohort 2). Subjects with existing diabetic ocular complications (ICD codes: E11.3, E12.3, E13.3, E14.3 H28.0, H35.8, and H36.0) in the year prior to the index date were excluded from cohort 1. In contrast, subjects diagnosed with DR (ICD code: H36.0) within 1 year of the index date were included in cohort 2. Subjects were followed using an on-treatment approach until the first occurrence of any of the following: 1) drug discontinuation, 2) outcome of interest, 3) death, or 4) the end of the study period.

### Outcomes

In cohort 1 (subjects without DR), the occurrence of DR was defined as in- or outpatient visits for DR (ICD code: H36.0). In cohort 2 (subjects with DR), progression of DR was defined as follows: 1) use of the procedure codes for panretinal photocoagulation (S5160) or vitrectomy (S5160 and S5121–22); or 2) the addition of diagnostic codes for vitreous hemorrhage (ICD-10 codes: H43.1 and H45.0), retinal detachment (ICD-10 codes: H33.0, H33.4, and H33.5), or neovascular glaucoma (ICD-10 codes: H40.5 and H40.88). The definition of DR progression was determined by the presence of diagnoses or treatments for proliferative stage DR based on the protocol of the Diabetic Retinopathy Clinical Research Network [22].

### Covariates

Baseline characteristics within 1 year prior to the index date (date of initial exposure) were used for analysis. We considered the following covariates as potential confounders: sex, age, comorbidities, use of drugs, body mass index (BMI), waist circumference, blood pressure, fasting glucose level, lipid levels, serum creatinine level, and lifestyle factors. A list of confounding variables with the corresponding ICD-10 codes, procedure codes, or Anatomical Therapeutic Chemical code is provided in S1 Table.

### Statistical analysis

Categorical variables are expressed as frequencies or percentages, and continuous variables as means with standard deviations. Propensity scores were matched in a 1:1 ratio using a caliper width equal to 0.25 of the standard deviation of the logits [23]. The probability of initiation of SGLT2i was estimated using a logistic regression model with confounder variables. In before-and-after propensity score matching, the covariate balance was calculated as standardized differences; significant imbalances were defined as standardized differences of ≥ 10% [24]. The time from the initiation of SGLT2i or DPP4i to events such as DR occurrence or progression was assessed using Kaplan–Maier plots and the log-rank test. Cox proportional hazards models were used to calculate the hazard ratios (HR) and 95% confidence intervals (CI) for each outcome. Additionally, we conducted subgroup analyses stratified by the major confounder variables. As a sensitivity analysis, we performed an intent-to-treat approach, where all subjects were followed from the index date until the outcome of interest, death, or end of the study period, whichever came first. All analyses were performed using SAS software (ver. 9.4, SAS Institute Inc., Cary, NC, USA), and survival curves using “survival” R package (ver. 2.42–6).

## Results

During the study period (2014 to 2016), 292,721 subjects without DR initiated therapy with a SGLT2i or a DPP4i (cohort 1), and 67,564 subjects with DR were identified (cohort 2) (Fig 1). Before propensity-score matching, subjects in cohort 1 initiating a SGLT2i were younger, more frequently female, and had a higher mean BMI and lower mean fasting glucose level than those initiating a DPP4i. In cohort 2, subjects initiating a SGLT2i were also younger, more frequently female, and had a higher mean BMI than those starting on a DPP4i; however, the mean fasting glucose levels were similar (S2 Table).

After matching, 20,715 and 4,663 pairs were identified in cohort 1 and 2, respectively (Fig 1). The baseline characteristics of the two groups were well-balanced (standardized differences for all variables were < 4%) (Table 1). The median follow-up duration was 234 ± 213 days for cohort 1, and 249 ± 206 days for cohort 2.

**Table 1:** Baseline characteristics in propensity score-matched cohorts of SGLT2i and DPP4i initiators.

	Cohort 1 (people without DR)					Cohort 2 (people with DR)				
	SGLT2i (N = 20,715)		DPP4i (N = 20,715)		Standardized difference (%)	SGLT2i (N = 4,663)		DPP4i (N = 4,663)		Standardized difference (%)
Age, mean (SD), years	54.1	(11.2)	54.1	(11.6)	0.2	58.3	(10.6)	57.9	(11.1)	3.5
Women	8,722	(42.1)	8,632	(41.7)	0.9	2,368	(50.8)	2,328	(49.9)	1.7
Index year										
2014	2,299	(11.1)	2,276	(11.0)	0.4	361	(7.7)	361	(7.7)	0.0
2015	6,972	(33.7)	6,915	(33.4)	0.6	1,726	(37.0)	1,746	(37.4)	0.9
2016	11,444	(55.2)	11,524	(55.6)	0.8	2,576	(55.2)	2,556	(54.8)	0.9
**Comorbidities**										
Myocardial infarction	418	(2.0)	441	(2.1)	0.8	134	(2.9)	129	(2.8)	0.6
CABG	7	(0.0)	7	(0.0)	0.0	8	(0.2)	11	(0.2)	1.4
PCI with stent	256	(1.2)	273	(1.3)	0.7	89	(1.9)	78	(1.7)	1.8
Unstable angina	713	(3.4)	750	(3.6)	1.0	315	(6.8)	290	(6.2)	2.2
Angina pectoris	2,503	(12.1)	2,533	(12.2)	0.4	963	(20.7)	968	(20.8)	0.3
Heart failure	886	(4.3)	842	(4.1)	1.1	314	(6.7)	310	(6.6)	0.3
Atrial fibrillation	379	(1.8)	384	(1.9)	0.2	94	(2.0)	98	(2.1)	0.6
Stroke	1,416	(6.8)	1,425	(6.9)	0.2	602	(12.9)	612	(13.1)	0.6
Peripheral artery disease	19	(0.1)	12	(0.1)	1.2	11	(0.2)	14	(0.3)	1.2
Chronic kidney disease	175	(0.8)	198	(1.0)	1.2	104	(2.2)	115	(2.5)	1.6
Diabetic neuropathy	2,805	(13.5)	2,797	(13.5)	0.1	1,836	(39.4)	1,818	(39.0)	0.8
Diabetic nephropathy	2,432	(11.7)	2,370	(11.4)	0.9	1,456	(31.2)	1,489	(31.9)	1.5
Severe hypoglycemia	301	(1.5)	305	(1.5)	0.2	187	(4.0)	196	(4.2)	1.0
Keto-/lactate acidosis	96	(0.5)	91	(0.4)	0.4	62	(1.3)	56	(1.2)	1.2
Cancer	1,701	(8.2)	1,656	(8.0)	0.8	596	(12.8)	613	(13.1)	1.1
Frailty (yes)	350	(1.7)	345	(1.7)	0.2	155	(3.3)	165	(3.5)	1.2
**Drugs**										
Glucose lowering drugs										
Metformin	15,757	(76.1)	15,740	(76.0)	0.2	3,716	(79.7)	3,666	(78.6)	2.6
Sulfonylurea	6,748	(32.6)	6,805	(32.9)	0.6	2,604	(55.8)	2,620	(56.2)	0.7
Thiazolidinediones	1,667	(8.0)	1,707	(8.2)	0.7	885	(19.0)	876	(18.8)	0.5
GLP-1 receptor agonists	32	(0.2)	27	(0.1)	0.6	27	(0.6)	23	(0.5)	1.2
Meglitinide	98	(0.5)	97	(0.5)	0.1	91	(2.0)	100	(2.1)	1.4
AGI	819	(4.0)	846	(4.1)	0.7	458	(9.8)	463	(9.9)	0.4
Insulin	1,622	(7.8)	1,614	(7.8)	0.1	557	(11.9)	588	(12.6)	2.0
CVD risk treatment										
Low dose acetylsalicylic acid	4,095	(19.8)	4,084	(19.7)	0.1	1,439	(30.9)	1,442	(30.9)	0.1
Statin therapy	12,975	(62.6)	12,989	(62.7)	0.1	3,391	(72.7)	3,417	(73.3)	1.3
ACE inhibitors	481	(2.3)	500	(2.4)	0.6	182	(3.9)	178	(3.8)	0.4
ARB	9,274	(44.8)	9,167	(44.3)	1.0	2,442	(52.4)	2,428	(52.1)	0.6
Dihydropyridines	3,606	(17.4)	3,632	(17.5)	0.3	928	(19.9)	953	(20.4)	1.3
Low ceiling diuretics	1,520	(7.3)	1,437	(6.9)	1.6	409	(8.8)	406	(8.7)	0.2
Beta blockers	3,063	(14.8)	3,076	(14.8)	0.2	824	(17.7)	832	(17.8)	0.4
Non-hydropyridines	473	(2.3)	466	(2.2)	0.2	171	(3.7)	158	(3.4)	1.5
High ceiling diuretics	714	(3.4)	700	(3.4)	0.4	289	(6.2)	321	(6.9)	2.8
Aldosterone antagonists	331	(1.6)	333	(1.6)	0.1	118	(2.5)	141	(3.0)	3.0
Warfarin	121	(0.6)	118	(0.6)	0.2	39	(0.8)	42	(0.9)	0.7
Receptor P2Y12 antagonists	1,348	(6.5)	1,359	(6.6)	0.2	601	(12.9)	617	(13.2)	1.0
Intravitreal injection						71	(1.5)	58	(1.2)	2.4
**Results of examination, mean (SD)**										
Body mass index (kg/m^2^)	27.5	(4.1)	27.5	(4.4)	0.3	26.7	(3.9)	26.8	(4.1)	1.4
Waist circumference (cm)	90.1	(9.9)	90.1	(13.7)	0.6	88.6	(9.6)	88.7	(10.0)	0.9
Systolic blood pressure (mmHg)	129.2	(15.0)	129.1	(15.2)	0.4	128.0	(14.9)	128.3	(15.0)	2.1
Diastolic blood pressure (mmHg)	80.1	(10.2)	80.1	(10.3)	0.1	77.4	(9.8)	77.7	(10.0)	3.4
Fasting glucose (mg/dL)	160.2	(56.5)	160.1	(55.0)	0.2	154.0	(55.8)	154.3	(56.3)	0.5
Total cholesterol (mg/dL)	199.8	(49.6)	199.3	(50.4)	1.0	180.6	(49.8)	180.9	(44.1)	0.6
HDL cholesterol (mg/dL)	49.7	(13.9)	49.7	(14.0)	0.1	50.2	(12.5)	50.1	(12.9)	1.2
LDL cholesterol (mg/dL)	113.3	(45.7)	112.6	(42.0)	1.7	98.5	(37.5)	99.2	(38.7)	1.9
Triglyceride (mg/dL)	200.8	(174.5)	200.0	(175.9)	0.4	165.4	(140.0)	165.8	(126.2)	0.3
Creatinine (mg/dL)	0.9	(0.7)	0.9	(0.3)	0.1	0.9	(0.4)	0.9	(0.3)	3.4
eGFR (mL/min/1.73 m^2^)	91.5	(25.4)	91.6	(26.8)	0.2	87.0	(24.5)	86.4	(24.2)	2.4
**Social history**										
Smoking status										
Non-smoker	11,076	(53.5)	11,058	(53.4)	0.2	2,876	(61.7)	2,823	(60.5)	2.3
Former smoker	4,328	(20.9)	4,290	(20.7)	0.5	972	(20.8)	977	(21.0)	0.3
Current smoker	5,311	(25.6)	5,367	(25.9)	0.6	815	(17.5)	863	(18.5)	2.7
Alcohol intake										
Abstinent	19,397	(93.6)	19,403	(93.7)	0.1	4,450	(95.4)	4,432	(95.0)	1.8
Low-medium	794	(3.8)	806	(3.9)	0.3	116	(2.5)	127	(2.7)	1.5
High	524	(2.5)	506	(2.4)	0.6	97	(2.1)	104	(2.2)	1.0
Physical activity										
Low	9,553	(46.1)	9,694	(46.8)	1.4	2,122	(45.5)	2,089	(44.8)	1.4
Medium	9,581	(46.3)	9,483	(45.8)	0.9	2,091	(44.8)	2,116	(45.4)	1.1
High	1,581	(7.6)	1,538	(7.4)	0.8	450	(9.7)	458	(9.8)	0.6

Abbreviations: ACE, angiotensin converting enzyme; AGI, alpha glucosidase inhibitor; ARB, angiotensin receptor blocker; CABG, coronary artery bypass grafting; CVD, cardiovascular disease; DPP4i, dipeptidyl peptidase-4 inhibitor; DR, diabetic retinopathy; eGFR, estimated glomerular filtration rate; GLP-1, glucagon-like peptide-1; HDL, high-density lipoprotein; LDL, low-density lipoprotein; PCI, percutaneous coronary intervention; SGLT2i, sodium-glucose cotransporter-2 inhibitor. Data are reported as numbers (percentages) unless otherwise stated.

**Fig 2:**
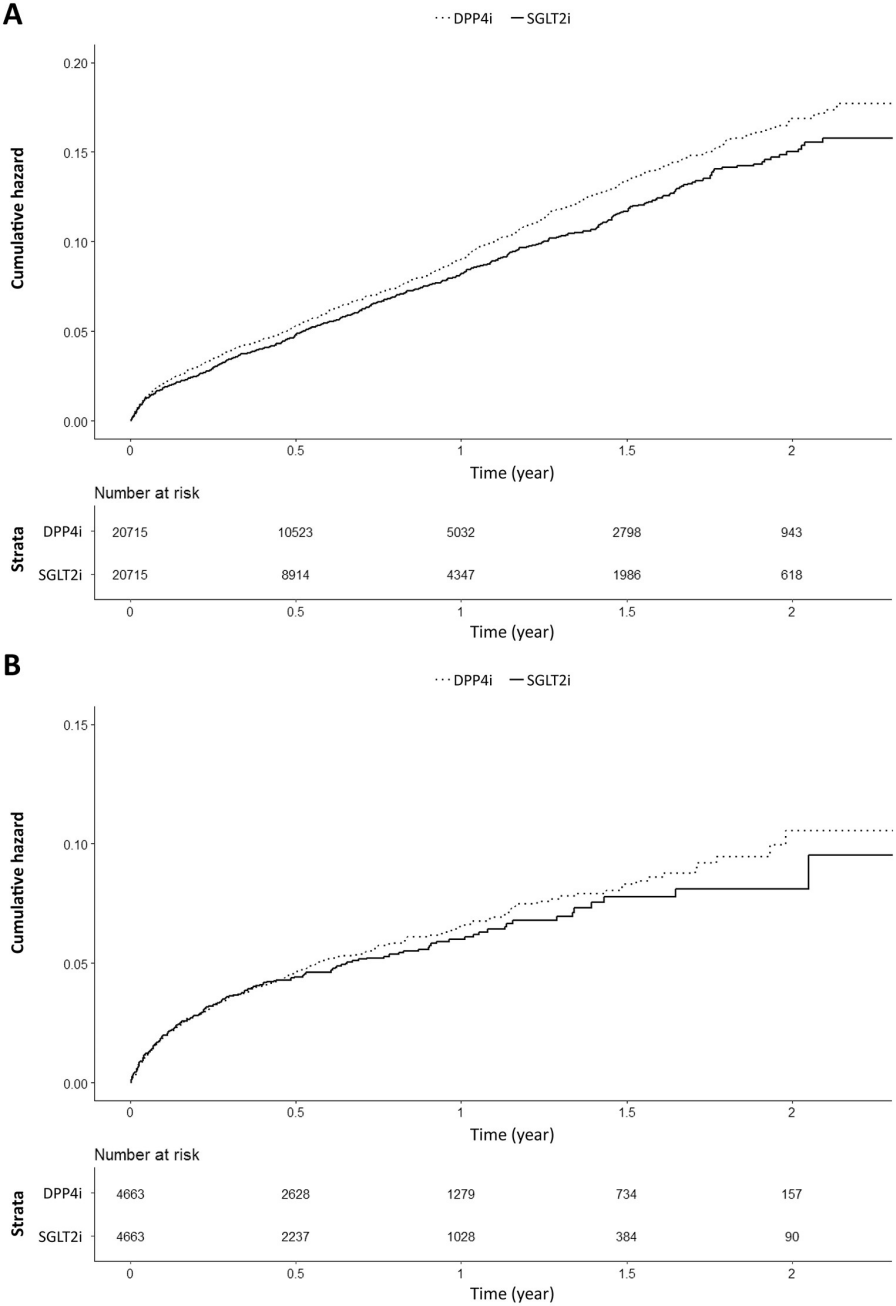
Cumulative incidence (A) and progression (B) of DR in SGLT2i initiators and DPP4i initiators. DPP4i, dipeptidyl peptidase-4 inhibitor; SGLT2i, sodium-glucose cotransporter-2 inhibitor.

**Fig 3:**
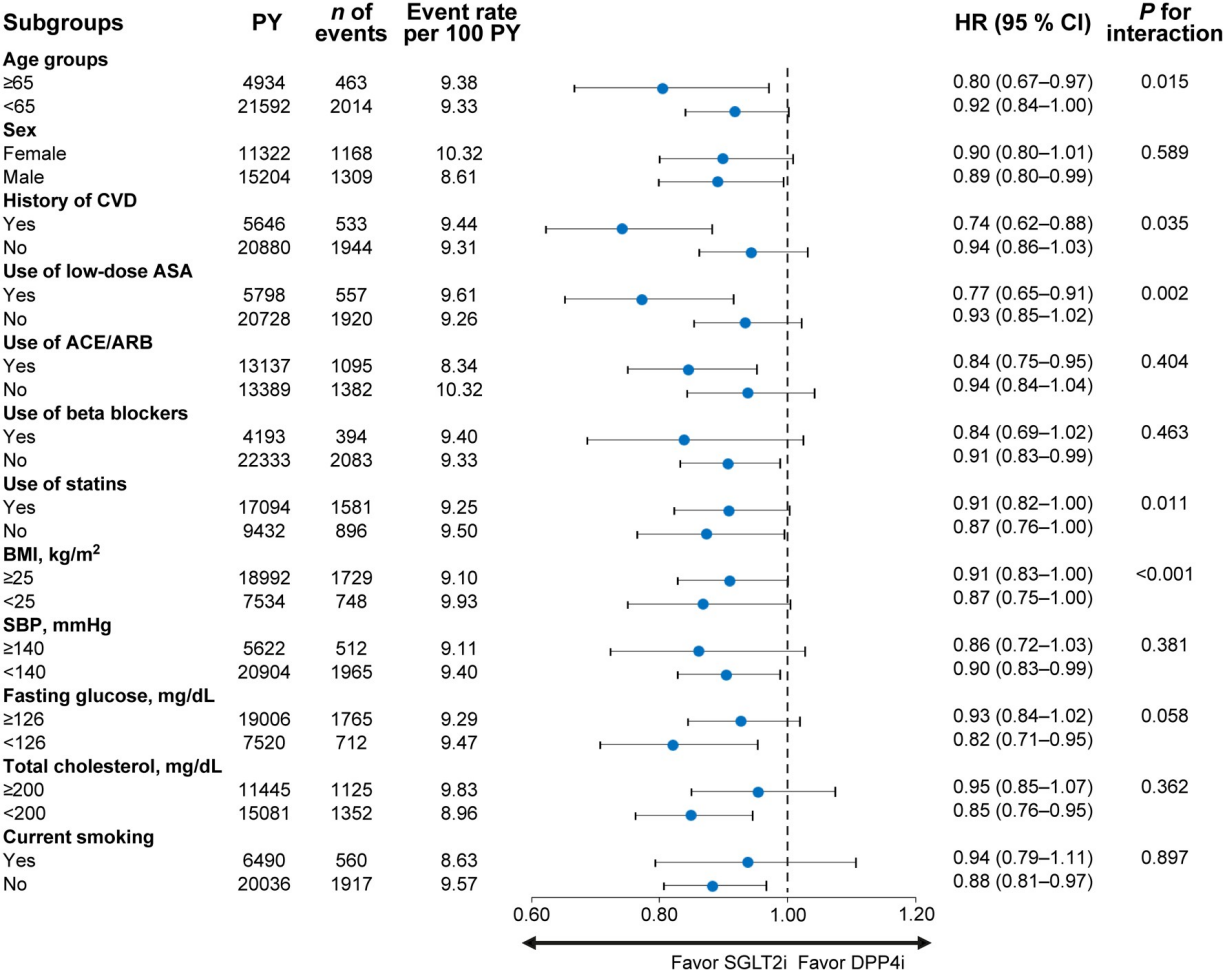
Subgroup analysis of HRs for DR in SGLT2i initiators and DPP4i initiators. ACE, angiotensin converting enzyme; ARB, angiotensin receptor blocker; ASA, acetylsalicylic acid; BMI, body mass index; CI, confidence interval; CVD, cardiovascular disease; DPP4i, dipeptidyl peptidase-4 inhibitor; HR, hazard ratio; PY, person-year; SBP, systolic blood pressure; SGLT2i, sodium-glucose cotransporter-2 inhibitor.

In cohort 1, SGLT2i initiators had a lower risk of DR than DPP4i initiators (HR 0.89, 95% CI 0.83–0.97, *P* = 0.006) (Table 2 and Fig 2). However, in the intent-to-treat analysis, there was no significant difference between SGLT2i initiators and DPP4i initiators (HR 0.95, 95% CI 0.89–1.02, *P* = 0.169) (S3 Table and S1 Fig). In subgroup analyses stratified by confounding factors, SGLT2i initiators had a lower risk of DR than DPP4i initiators, especially in those with a history of cardiovascular diseases, those treated with low-dose acetylsalicylic acid (ASA), those with fasting glucose < 126 mg/dL, and those systolic blood pressure < 140 mmHg (Fig 3). We also performed a sensitivity analysis by investigating the occurrence of DR using the prevalence of ICD code-defined DR among subjects that ever had the procedure codes for fundoscopy or fundus photograph (E6660, E6670, and E6681). The results revealed similar tendency without statistical significance due to declined number of patients (Table 3).

**Table 2:** HRs for the occurrence and progression of DR in propensity score-matched analyses.

Drugs	Cohort 1 (people without DR)				Cohort 2 (people with DR)			
	No. of people	PY	No. of events	Event rate (per 100 PY)	No. of people	PY	No. of events	Event rate (per 100 PY)
SGLT2i	20,715	12,295	1,098	8.93	4,663	2,899	205	7.07
DPP4i	20,715	14,231	1,379	9.69	4,663	3,455	241	6.98
HR (95% CI; *P* value)	0.89 (0.83–0.97; 0.006)				0.94 (0.78–1.13; 0.506)			

Abbreviations: CI, confidence interval; DPP4i, dipeptidyl peptidase-4 inhibitor; DR, diabetic retinopathy; HR, hazard ratio; PY, person-years; SGLT2i, sodium-glucose cotransporter-2 inhibitor.

**Table 3:** Sensitivity analysis for the prevalence of ICD code-defined DR among the subjects that ever had the procedure codes for fundus examination.

Drugs	Cohort 1 (people without DR)			
	No. of people	PY	No. of events	Event rate (per 100 PY)
SGLT2i	4,543	2,658	292	10.98
DPP4i	4,543	3,111	343	11.03
HR (95% CI; *P* value)	0.98 (0.84–1.15; 0.842)			

Abbreviations: CI, confidence interval; DPP4i, dipeptidyl peptidase-4 inhibitor; DR, diabetic retinopathy; HR, hazard ratio; PY, person-years; SGLT2i, sodium-glucose cotransporter-2 inhibitor

In cohort 2, the risk of DR progression was not significantly different between SGLT2i initiators and DPP4i initiators (HR 0.94, 95% CI 0.78–1.13, *P* = 0.506) (Table 2 and Fig 2). The intention-to-treat analysis also yielded results consistent with the main findings (HR 0.98, 95% CI 0.83–1.15, *P* = 0.803) (S3 Table and S1 Fig).

## Discussion

The key findings of this study are that the SGLT2i initiators had a lower risk of DR than the DPP4i initiators with 11% reduction in the incidence of DR with SGLT2i compared to DPP4i. No significant difference was noted in the aspect of DR progression between SGLT2i and DPP4i initiators.

Use of a SGLT2i is reportedly associated with a lower risk of cardiovascular mortality in people with type 2 diabetes [3, 6, 25]. Management of cardiovascular disease is critical as this is the leading cause of mortality and morbidity in type 2 diabetic patients, but management of microvascular complications, such as DR, is also vital as severe visual impairment can reduce patients’ quality of life. DR is a cause of severe visual impairment in the working-age population worldwide [26–28]. Recent estimates suggest that the number of people with DR will increase from 127 million in 2010 to 191 million in 2030 [26]. Retinal neovascularization and its accompanying complications, such as vitreous hemorrhage, tractional retinal detachment, and neovascular glaucoma in the proliferative stage, as well as diabetic edema, can result in severe visual loss in DR patients [17]. Current treatment modalities have limitations in terms of their ability to restore already-impaired vision and their high cost [18].

Use of DPP4is has increased significantly over the past decade, but their effect on DR is still controversial [29–32]. As both SGLT2is and DPP4is are relatively new antidiabetic medications, it is presently impossible to adequately assess their long-term effects on the risk of microvascular events, such as DR. However, in large-scale trials using renal outcomes, SGLT2i exerted a protective effect relatively early, within 3–4 years [33, 34]. A recently published subgroup analysis of EMPA-REG OUTCOME in Asian patients reported that renal functions was improved from 12 weeks which effects were maintained through 3 years [34]. This early effect of SGLT2is in nephropathy could support the short-term effect in DR of this study, as both retinopathy and nephropathy are similar microvascular complications of diabetes [16]. Although the lower HR in the intent-to-treat analysis was not statistically significant, the results should be interpreted cautiously as bias from dilution effect can exist [35].

Retinal pericytes are reported to express SGLT2, so that SGLT2is may directly protect against retinal pericyte swelling and loss and prevent hyperperfusion in the retina under high-glucose conditions, which are critical steps in the pathogenesis of early-stage DR [36–38]. Other established multifactorial effects of SGLT2i may contribute to retinal protection. Improved glycemic control, lowering of blood pressure and retinal capillary blood flow, blocking of the renin-angiotensin system, and preventing changes to the structure of the retinal arterioles are all plausible protective mechanisms [39–41]. With these protective effects of SGLT2is, our study showed that a lower risk of DR occurrence in the following groups; 1) those with a history of cardiovascular diseases, 2) those treated with low-dose acetylsalicylic acid (ASA), 3) those with fasting glucose < 126 mg/dL, and 4) those systolic blood pressure < 140 mmHg.

The protective effect of SGLT2is in patients with history of CVD can be explained by strict regulation of blood pressure and glucose levels. Since CVD is well known as the principal cause of death and disability among patients with diabetes, it is safe to assume that tight control of blood pressure and metabolic factors such as HbA1c and dyslipidemia would have been recommended by physicians and strictly followed by these patients [4, 9, 42]. For these reasons, it is believed that the additive effect of modification of systemic factors and SGLT2is was beneficial to patients with history of CVD.

Similar hypothesis ca be applied to those with fasting glucose < 126 mg/dL and those with systolic blood pressure < 140 mmHg. Modification of systemic risk factors such as hyperglycemia and hypertension are critical to minimizing risk of onset and progression of DR. Our results are consistent with the results of UKPDS (< 150 mmHg) and ACCORD (< 140 mmHg) [43, 44]. It should also be pointed out that fasting glucose level 126mg/dl is considered as cut off value for detecting diabetes by the American Diabetes Association and World Health Organization, which means hyperglycemia was well under control in the groups with fasting glucose < 126 mg/dl in our study. Based on these, it is suggested that the benefit of SGLT2i for reducing risk of retinopathy occurrence can be intensified in patients with better glycemic control and lower blood pressure.

For low-dose ASA, it is known to prevent the development of acellular capillaries due to its anti-inflammatory and anti-thrombotic effect in preclinical studies and clinical studies [45–47]. However, there is no large clinical study of aspirin which shows its protective effects on the occurrence of retinopathy so far. It can be assumed that the protective effect of SGLT2i against retinal pericyte swelling and loss was potentiated with anti-inflammatory and anti-thrombotic effect of aspirin only in diabetic patients without DR, since pericyte cell death lead to endothelial cell proliferation and microaneurysm development.

DR progression did not differ significantly between SGLT2i initiators and DPP4i initiators. Glycemic control and a reduction in blood pressure decrease the risk of DR progression and the minimal difference of glycemic control between SGLT2is and DPP4, in our study, is may be insufficient to affect DR progression.[43, 48–50]. However, intensive glycemic control did not reduce the need of retinal photocoagulation or vitrectomy for advanced DR, suggesting no significant effect in advanced microvascular complications [51]. Recently, Inzucchi et al. [52] reported DR outcomes with empagliflozin versus placebo in the EMPA-REG OUTCOME Trial, which used similar outcomes such as retinal photocoagulation, vitreous hemorrhage, and administration of intravitreal agents as our study. They concluded empagliflozin was not associated with an increased risk of retinopathy compared with placebo in the EMPA-REG OUTCOME trial in patients with type 2 diabetes [52]. They suggested that one of the reasons for this result was because the nature and severity of retinopathy was not collected. The same hypothesis can also explain our results. Unlike cohort 1 which had clear outcome from no retinopathy to retinopathy, the outcome of cohort 2 was determined by the presence of diagnoses or treatments for proliferative stage DR. While DR at baseline was coded using ICD-10 codes, the severity of DR was not collected in our study. Furthermore, lack of HbA1c and duration of diabetes in each treatment group also can be the reason that SGLT2is had no effect on DR progression in our study. Thus, the effect of SGLT2is on DR progression requires further studies with a longer follow-up period and a larger population. There are currently clinical trials attempting to investigate the effect of SGLT2i on the DR progression as well as diabetic macular edema [53].

### Limitations

Our findings should be examined within the context of several potential limitations. The lack of glycosylated hemoglobin (HbA1c) data is one of the major limitations of this study as strict glycemic control delays DR progression [43, 54, 55]. We attempted to overcome this by adjusting for fasting glucose levels, as the HbA1c data was not included in the NHIS health screening protocol. There are also reports of no significant differences in glycemic control among different glucose-lowering agents, while some studies reported that the reduction in the HbA1c level differed between SGLT2i and DPP4i according to the baseline HbA1c levels [56–58]. The UK Prospective Diabetes Study, a landmark study of the effect of glycemic control on DR in patients with type 2 diabetes, reported that each 1% decrement in HbA1c level led to a 31% reduction in the incidence of DR [59]. Because SGLT2is resulted in a slightly greater reduction in the HbA1c level than DPP4is (weighted mean difference of 0.13% in systemic reviews and a meta-analysis [56]), use of a SGLT2i rather than a DPP4i might explain 4% of the 11% reduction in the incidence of DR.

The lack of bevacizumab data is also an important limitation, as intravitreal anti-VEGF injections can ameliorate DR [22, 60]. Bevacizumab is an anti-VEGF agent frequently used to treat DR-associated complications. In this study, use of bevacizumab could not be identified as it is not covered by the NHIS due to its off-label use in Korea. However, there were no differences in the frequencies of intravitreal injections of other agents (ranibizumab, aflibercept, triamcinolone, and dexamethasone implant) between the two cohorts.

The lack of statistical significance at the sensitivity analysis is also an important limitation. By defining the occurrence of DR as the number of new ICD code-defined DR per the number of diabetic subjects that ever received fundus examination, the number of included people has declined from 20,175 to 4,543 respectively. The tendency of lower risk of DR in SGLT2i initiators than the DPP4i initiators was not significant in the sensitivity analysis. Analyses with larger number of patients who received fundus examination would be helpful to confirm the effect of SGLT2i concerning the prevention of DR.

The relatively short duration of follow-up is another limitation to be noted. This study should be cautiously interpreted in the aspect of short-term effect of studied medications, as showed renal outcomes reporting improved kidney functions within several weeks [34]. Further studies with longer duration are needed to evaluate long-term effect of SGLT2is.

This study had several other minor limitations. First, the occurrence or progression of DR was defined using diagnostic or procedure codes in the NHIS database. Although provision of a diagnostic or procedure code is mandatory in the NHIS, they may be inaccurate. Second, selection bias may have been caused by restriction of the study population to those with available health-screening data. Nevertheless, this allowed adjustment for major confounding factors, such as fasting glucose levels and lipid profiles.

## Conclusions

In conclusion, this real-world cohort study showed that the use of a SGLT2i was associated with lower risk of DR occurrence but was not different in the risk of DR progression compared to a DPP4i. These effect of SGLT2i against DR is likely mediated in part by its glucose-lowering activity, as well as other mechanisms associated with lowered blood pressure. Randomized controlled studies with longer duration are needed to confirm the benefits of SGLT2i concerning the prevention of DR.

## Supporting information

S1 Fig Cumulative DR incidence (A) and progression (B) in SGLT2i initiators and DPP4i initiators in the intent-to-treat analysis. DPP4i, dipeptidyl peptidase-4 inhibitor; SGLT2i, sodium-glucose cotransporter-2 inhibitor.
(TIF)

S1 Table List of diagnoses, treatments, and procedures, and their corresponding codes. (PDF)

S2 Table Baseline characteristics in cohorts of SGLT2i and DPP4i initiators before propensity score-matching. (PDF)

S3 Table Hazard ratios for the occurrence and progression of DR in propensity score-matched analyses (intent-to-treat analysis). (PDF)
