# Supplementary figures and images for: Effects of sodium-glucose cotransporter-2 inhibitors and dipeptidyl peptidase-4 inhibitors on diabetic retinopathy and its progression: A real-world Korean study
Source: PLoS One. 2019 Oct 28;14(10):e0224549. doi: 10.1371/journal.pone.0224549 (PMC6816558; doi:10.1371/journal.pone.0224549)

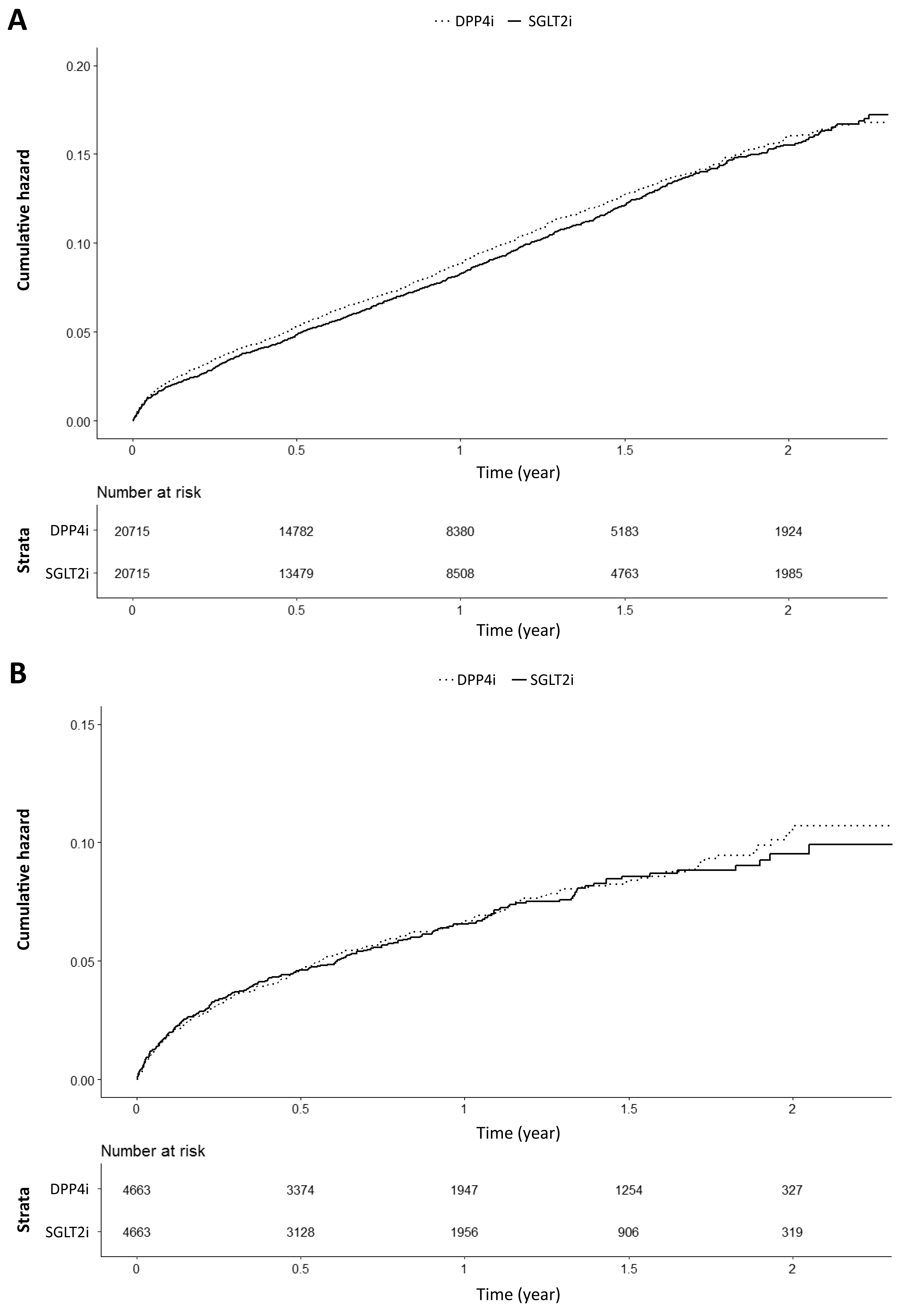

Supplement: S1 Fig — DPP4i, dipeptidyl peptidase-4 inhibitor; SGLT2i, sodium-glucose cotransporter-2 inhibitor. (TIF) [file pone.0224549.s001.tif]
