# Supplementary material for: Effects of sodium-glucose cotransporter-2 inhibitors and dipeptidyl peptidase-4 inhibitors on diabetic retinopathy and its progression: A real-world Korean study
Source: PLoS One. 2019 Oct 28;14(10):e0224549. doi: 10.1371/journal.pone.0224549 (PMC6816558; doi:10.1371/journal.pone.0224549)
Supplement: S1 Table — (PDF) [file pone.0224549.s002.pdf]

S1Table. List of diagnoses, treatments, and procedures, and their corresponding codes.

**Diagnoses**	
Myocardial infarction	I21, I22
CABG	O1640-9, OA640-2, OA647-9
PCI with stent	M6551-2, M6561-7
Unstable angina	I20.0
Angina pectoris	I20.1, I20.8, I20.9
Heart failure	I50
Atrial fibrillation	I48
Stroke	I60-I66, G45
Peripheral artery disease	I70-79 and M6597, M6605, M6613, M6632, M6620, O0161-O0171, O1643-4, O1645-6, I70.2-3, I70.9, I73.1, I73.8-9
Chronic kidney disease	N18
Diabetic neuropathy	G59.0, G63.2, G99.0, E10.4, E11.4, E12.4, E13.4, E14.4
Diabetic nephropathy	N08.3, E10.2, E11.2, E12.2, E13.2, E14.2
Diabetic retinopathy	E11.3, E12.3, E13.3, E14.3 H28.0, H35.8, H36.0
Severe hypoglycemia	E10.0, E11.0, E12.0, E13.0, E14.0, E11.6A, E16.0-2
Keto-/lactate acidosis	E10.1, E11.1, E12.1, E13.1, E14.1, E87.2
Cancer	C00-C99
**Medications**	
Glucose-lowering drugs	
SGLT2i	A10BK01, A10BK02, A10BK03, A10BD15, A10BD16, A10BD20, A10BX
DPP4i	A10BH, A10BD07, A10BD08, A10BD09, A10BD10, A10BD11, A10BD13, A10BD18
Metformin	A10BA
Sulfonylurea	A10BB, A10BD02, A10BD04, A10BD06
Thiazolidinediones	A10BG, A10BD03, A10BD04, A10BD05, A10BD06, A10BD09, A10BD
GLP-1 receptor agonists	A10BJ
AGI	A10BF, A10BD
Meglitinide	A10BX02, A10BX03, A10BX08, A10BD14, A10BD
Insulin	A10A
short-acting	A10AB
intermediate-acting	A10AC
premixed insulin	A10AD
long-acting	A10AE
Statin	C10AA
Antihypertensives	
ACE inhibitors	C09A, C09B
ARB	C09C, C09D (exclude C09DX04)
Dihydropyridines	C08C
Low ceiling diuretics	C03A, C03B
Beta blockers	C07
Non-hydropyridines	C08D
High ceiling diuretics	C03C
Aldosterone antagonists	C03DA
Low dose acetylic salicylic acid	B01AC06
Warfarin	B01AA03
Receptor P2Y12 antagonists	B01AC04, B01AC05, B01AC22, B01AC24

ACE, angiotensin converting enzyme; AGI, alpha glucosidase inhibitor; ARB, angiotensin receptor blocker; CABG, coronary artery bypass grafting; DPP4i, dipeptidyl peptiase-4 inhibitor; GLP-1, glucagon-like peptide-1; PCI, percutaneous coronary intervention; SGLT2i: sodium-glucose cotransporter-2 inhibitor
