# Supplementary material for: Effects of sodium-glucose cotransporter-2 inhibitors and dipeptidyl peptidase-4 inhibitors on diabetic retinopathy and its progression: A real-world Korean study
Source: PLoS One. 2019 Oct 28;14(10):e0224549. doi: 10.1371/journal.pone.0224549 (PMC6816558; doi:10.1371/journal.pone.0224549)
Supplement: S2 Table — (PDF) [file pone.0224549.s003.pdf]

S2 Table. Baseline characteristics in cohorts of SGLT2i and DPP4i initiators before propensity score-matching.

	Cohort 1 (people without DR)					Cohort 2 (people with DR)				
	SGLT2i (N = 20,725)		DPP4i (N = 271,996)		Standardized difference (%)	SGLT2i (N = 4,683)		DPP4i (N = 62,881)		Standardized difference (%)
Age, mean (SD), years	54.1	(11.2)	58.4	(11.6)	37.6	58.2	(10.7)	63.0	(10.6)	45.2
Women	8,728	(42.1)	99,842	(36.7)	11.1	2,380	(50.8)	29,098	(46.3)	9.1
Index year										
2014	2,299	(11.1)	32,970	(12.1)	3.2	361	(7.7)	6,505	(10.3)	9.2
2015	6,974	(33.7)	115,786	(42.6)	18.4	1,730	(36.9)	29,699	(47.2)	21.0
2016	11,452	(55.3)	123,240	(45.3)	20.0	2,592	(55.3)	26,677	(42.4)	26.1
**Comorbidities**										
Myocardial infarction	418	(2.0)	5,298	(1.9)	0.5	134	(2.9)	1,939	(3.1)	1.3
CABG	7	(0.0)	175	(0.1)	1.4	8	(0.2)	95	(0.2)	0.5
PCI with stent	256	(1.2)	2,575	(0.9)	2.8	89	(1.9)	1,154	(1.8)	0.5
Unstable angina	713	(3.4)	8,303	(3.1)	2.2	318	(6.8)	3,880	(6.2)	2.5
Angina pectoris	2,503	(12.1)	30,752	(11.3)	2.4	968	(20.7)	12,443	(19.8)	2.2
Heart failure	886	(4.3)	11,783	(4.3)	0.3	314	(6.7)	4,752	(7.6)	3.3
Atrial fibrillation	379	(1.8)	5,312	(2.0)	0.9	94	(2.0)	1,834	(2.9)	5.9
Stroke	1,416	(6.8)	23,649	(8.7)	7.0	603	(12.9)	10,177	(16.0)	9.0
Peripheral artery disease	19	(0.1)	324	(0.1)	0.8	11	(0.2)	359	(0.6)	5.3
Chronic kidney disease	175	(0.8)	4,815	(1.8)	8.2	104	(2.2)	3,959	(6.3)	20.3
Diabetic neuropathy	2,797	(13.5)	37,520	(13.8)	0.9	1,850	(39.5)	23,699	(37.7)	3.7
Diabetic nephropathy	2,436	(11.8)	25,663	(9.4)	7.5	1,467	(31.3)	16,569	(26.3)	11.0
Severe hypoglycemia	301	(1.5)	4,696	(1.7)	2.2	188	(4.0)	2,772	(4.4)	2.0
Keto-/lactate acidosis	96	(0.5)	1,158	(0.4)	0.6	64	(1.4)	692	(1.1)	2.4
Cancer	1,701	(8.2)	26,537	(9.8)	5.4	597	(12.7)	8,910	(14.2)	4.2
Frailty (yes)	350	(1.7)	8,172	(3.0)	8.7	158	(3.4)	2,955	(4.7)	6.7
**Drugs**										
Glucose lowering drugs										
Metformin	15,767	(76.1)	163,917	(60.3)	34.4	3,736	(79.8)	45,135	(71.8)	18.8
Sulfonylurea	6,752	(32.6)	104,879	(38.6)	12.5	2,616	(55.9)	39,262	(62.4)	13.4
Thiazolidinediones	1,669	(8.1)	17,868	(6.6)	5.7	874	(19.1)	8,122	(12.9)	16.9

GLP-1 receptor agonists	35	(0.2)	48	(0.0)	5.0	37	(0.8)	46	(0.1)	11.0
Meglitinide	99	(0.5)	1,757	(0.6)	2.3	91	(1.9)	1,716	(2.7)	5.2
AGI	821	(4.0)	11,793	(4.3)	1.9	458	(9.8)	6,927	(11.0)	4.1
Insulin	1,625	(7.8)	29,364	(10.8)	10.2	1,484	(31.7)	18,660	(29.7)	4.4
CVD risk treatment										
Low dose acetylic salicylic acid	4,097	(19.8)	62,056	(22.8)	7.4	1,443	(30.8)	22,074	(35.1)	9.1
Statin therapy	12.982	(62.6)	152,156	(55.9)	13.7	3,405	(72.7)	41,768	(66.4)	13.7
ACE inhibitors	481	(2.3)	6,635	(2.4)	0.8	182	(3.9)	2,841	(4.5)	3.1
ARB	9,280	(44.8)	114,533	(42.1)	5.4	2,452	(52.4)	32,600	(51.8)	1.0
Dihydropyridines	3,607	(17.4)	55,562	(20.4)	7.7	928	(19.8)	15,730	(25.0)	12.5
Low ceiling diuretics	1,521	(7.3)	19,513	(7.2)	0.6	410	(8.8)	5,665	(9.0)	0.9
Beta blockers	3,064	(14.8)	39,564	(14.6)	0.6	827	(17.7)	12,396	(19.7)	5.3
Non-hydropyridines	473	(2.3)	6,344	(2.3)	0.3	171	(3.7)	2,352	(3.7)	0.5
High ceiling diuretics	714	(3.4)	14,662	(5.4)	9.5	289	(6.2)	6,041	(9.6)	12.8
Aldosterone antagonists	332	(1.6)	5,622	(2.1)	3.5	118	(2.5)	1,861	(3.0)	2.7
Warfarin	121	(0.6)	2,105	(0.8)	2.3	39	(0.8)	736	(1.2)	3.4
Receptor P2Y12 antagonists	1,348	(6.5)	18,794	(6.9)	1.6	603	(12.9)	7,975	(12.7)	0.6
Intravitreal injection						71	(1.5)	1,082	(1.7)	1.6
**Results of examination, mean (SD)**										
Body mass index (kg/m^2)	27.5	(4.2)	25.67.6	(3.6)	48.6	26.7	(3.9)	24.9	(3.4)	51.1
Waist circumference (cm)	90.1	(10.0)	86.7	(9.3)	34.6	88.7	(9.7)	85.8	(8.8)	34.5
Systolic blood pressure (mmHg)	129.2	(15.0)	128.8	(15.3)	2.6	128.0	(14.9)	128.3	(15.4)	1.8
Diastolic blood pressure (mmHg)	80.1	(10.2)	79.1	(10.1)	9.4	77.4	(9.8)	76.6	(9.8)	7.6
Fasting glucose (mg/dL)	160.2	(56.5)	164.4	(61.0)	7.2	154.1	(56.0)	153.8	(58.0)	0.6
Total cholesterol (mg/dL)	199.8	(49.6)	198.9	(50.9)	1.9	180.6	(49.7)	179.6	(46.4)	2.2
HDL cholesterol (mg/dL)	49.7	(13.9)	50.1	(14.2)	2.7	50.2	(12.5)	50.1	(13.9)	1.2
LDL cholesterol (mg/dL)	113.3	(45.7)	113.4	(49.1)	0.2	98.5	(37.5)	99.6	(42.0)	2.7
Triglyceride (mg/dL)	200.7	(174.4)	189.8	(163.3)	6.4	165.5	(139.9)	155.1	(114.5)	8.1
Creatinine (mg/dL)	0.9	(0.7)	0.9	(0.7)	6.7	0.9	(0.4)	1.0	(0.9)	22.6
eGFR (mL/min/1.73 m^2)	91.5	(25.4)	88.2	(25.5)	13.1	87.1	(24.5)	80.9	(27.7)	23.8
**Social history**										
Smoking status										
Non-smoker	11,083	(53.5)	139,615	(51.3)	4.3	2,888	(61.7)	38,730	(61.6)	0.2
Former smoker	4,330	(20.9)	60,524	(22.3)	3.3	974	(20.8)	13,888	(22.1)	3.1

Current smoker	5,312	(25.6)	71,857	(26.4)	1.8	821	(17.5)	10,263	(16.3)	3.2
Alcohol intake										
Abstinent	19,405	(93.6)	254,633	(93.6)	0.1	4,469	(95.4)	60,543	(96.3)	4.3
Low-medium	796	(3.8)	10,765	(4.0)	0.6	117	(2.5)	1,442	(2.3)	1.3
High	524	(2.5)	6,598	(2.4)	0.7	97	(2.1)	896	(1.4)	4.9
Physical activity										
Low	9,557	(46.1)	128,536	(47.3)	2.3	2,130	(45.5)	29,408	(46.8)	2.6
Medium	9,586	(46.3)	120,352	(44.2)	4.0	2,103	(44.9)	27,043	(43.0)	3.8
High	1,582	(7.6)	23,108	(8.5)	3.2	450	(9.6)	6,430	(10.2)	2.1

ACE, angiotensin converting enzyme; AGI, α-glucosidase inhibitor; ARB, angiotensin receptor blocker; CABG, coronary artery bypass grafting; CVD, cardiovascular disease; DPP4i, dipeptidyl peptidase-4 inhibitor; DR, diabetic retinopathy; eGFR, estimated glomerular filtration rate; GLP-1, glucagon-like peptide-1; HDL, high-density lipoprotein; LDL, low-density lipoprotein; PCI, percutaneous coronary intervention; SGLT2i, sodium-glucose cotransporter-2 inhibitor. Data are reported as numbers (percentages) unless otherwise stated.
