# Supplementary material for: Effects of sodium-glucose cotransporter-2 inhibitors and dipeptidyl peptidase-4 inhibitors on diabetic retinopathy and its progression: A real-world Korean study
Source: PLoS One. 2019 Oct 28;14(10):e0224549. doi: 10.1371/journal.pone.0224549 (PMC6816558; doi:10.1371/journal.pone.0224549)
Supplement: S3 Table — (PDF) [file pone.0224549.s004.pdf]

S3 Table. Hazard ratios for the occurrence and progression of DR in propensity score-matched analyses (intent-to-treat analysis).

Drugs	Cohort 1 (people without DR)				Cohort 2 (people with DR)			
	No. of people	PY	No. of events	Event rate (per 100 PY)	No. of people	PY	No. of events	Event rate (per 100 PY)
SGLT2i	20,715	19,019	1,608	8.45	4,663	4,229	272	6.43
DPP4i	20,715	20,080	1,768	8.80	4,663	4,562	292	6.40
HR (95% CI; *P* value)	0.95 (0.89 – 1.02; 0.169)				0.98 (0.83 – 1.15; 0.803)			

CI, confidence interval; DPP4i, dipeptidyl peptidase-4 inhibitor; DR, diabetic retinopathy; HR, hazard ratio; PY, person-years; SGLT2i, sodium-glucose cotransporter-2 inhibitor.
